# The complete plastid genome sequence of *Vaccinium japonicum* (Ericales: Ericaceae), a deciduous broad-leaved shrub endemic to East Asia

**DOI:** 10.1080/23802359.2021.1935351

**Published:** 2021-06-07

**Authors:** Won-Bum Cho, Eun-Kyeong Han, In-Su Choi, Dong Chan Son, Gyu Young Chung, Jung-Hyun Lee

**Affiliations:** a Department of Plant Variety Protection, Korea Forest Seed and Variety Center, Chungju, Republic of Korea; b Department of Biological Sciences and Biotechnology, Chonnam National University, Gwangju, Republic of Korea; c School of Life Sciences, Arizona State University, Tempe, AZ, USA; d Department of Division of Forest Biodiversity and Herbarium, Korea National Arboretum, Pocheon, Republic of Korea; e Division of Horticulture and Medicinal Plant, Andong National University, Andong, Republic of Korea; f Department of Biology Education, Chonnam National University, Gwangju, Republic of Korea

**Keywords:** complete plastid genome, Ericaceae, *Vaccinium japonicum*, phylogenetic analysis, ndhF

## Abstract

We here sequenced the complete plastid genome (plastome) of *Vaccinium japonicum* (Ericaceae), a deciduous broad-leaved shrub endemic to East Asia. This species has considerable practical economic value. The plastome of *V. japonicum* is assembled as a single contig (187,213 bp). A large single copy (104,637 bp) and a small single copy (3,000 bp) of the genome are separated by a pair of inverted repeats (39,788 bp). The genome consists of 135 genes, which include 88 protein coding, eight ribosomal RNA, and 39 transfer RNA genes. The plastome of *V. japonicum* is similar to that of *Vaccinium macrocarpon* in gene content and order. Our phylogenetic analysis revealed the phylogenetic position of *V. japonicum* in a highly supported clade of the genus *Vaccinium* together with other four congeners, *V. bracteatum*, *V. vitis-idaea*, *V. uliginosum* and *V. macrocarpon.*

*Vaccinium* L., the largest genus within the blueberry tribe (Vaccinieae Rchb.) in the family Ericaceae, consists of approximately 450 species that are widely distributed globally (Vander Kloet and Dickinson 2009; Kim et al. 2020). Owing to the economic, agricultural, and medical importance, several plastid genomes (plastomes) of this genus have been sequenced (Fajardo et al. 2013). Moreover, several studies have provided evidence for powerful phylogenetic utilities and, particularly, noteworthy evolutionary evidence for early-diverging *Vaccinium* (Kim et al. 2019; Kim et al. 2020). Nonetheless, there is limited understanding of the evolution of species of the genus *Vaccinium*, especially with regard to the East Asian species.

*Vaccinium japonicum* Miq. is a deciduous broad-leaved shrub belonging to the section *Oxycoccoides* and is widely distributed in the warm-temperate regions of East Asia, including China, Korea (Jeju Island), and Japan. The main characteristics of this species include corolla divided nearly to the base and margin strongly revolute, and morphologically indistinct from *Vaccinium erythrocarpum* Michx. distributed in North America. For these reasons, *V. japonicum* is sometimes treated as a subspecies of *V. erythrocarpum* (Vander Kloet and Bohm 1991; Tamada 2004), which still under taxonomic controversy. *Vaccinium japonicum* consists of three varieties: *V. japonicum* var. *japonicum* is restricted to Korea (Jeju Island; Lee 2003; Kong et al. 2014) and Japan (Yamazaki 1993), *V. japonicum* var. *sinicum* (Nakai) Rehder to China (Ruizheng and Stevens 2005), and *V. japonicum* var. *lasiostemon* Hayata to Taiwan (Boufford et al. 2003). The complete plastome sequence of *V. japonicum* var. *japonicum* can provide crucial clues regarding the evolutionary process of the genus, and solve taxonomic controversies of inter/intra species within the section *Oxycoccoides*. We here sequenced and characterized a complete plastome from *V. japonicum* endemic to East Asia.

*Vaccinium japonicum* samples were collected from Jeju Island, South Korea (33°17′59.3ʺN, 126°35′02.7ʺE). The voucher specimen (Lee. 2007016) was stored in the Korea National Arboretum (sdclym@korea.kr). The total genomic DNA was sequenced on MGI-seq 2000 platform (LAS, Seoul, Korea) following manufacturer’s protocols. It generated 57,912,004 raw reads (150 bp paired-end). The plastome was assembled using NOVOPlasty 4.1 (Dierckxsens et al. 2017), with the *V. macrocarpon* rbcL gene sequence (Fajardo et al. 2013; JQ757046) as the seed. The assembled plastome was checked using Geneious 10.2.3 (Kearse et al. 2012) by mapping 590,424 reads, resulting in a coverage of 150x. The annotation was separately performed using Geneious and was manually corrected for the start and stop codons as well as for the intron/exon boundaries. The annotated plastome sequence was deposited in GenBank (accession number: MW006668). To construct the phylogenetic tree, plastomes of 13 species from the NCBI database were downloaded. The alignments were performed using MAFFT (Katoh and Toh 2010). The maximum likelihood (ML) analysis was performed with RAxML v.8.0 (Stamatakis, 2014) using default parameters and 1000 bootstrap replicates. For the RAxML tree, the general time-reversible (GTR) model of nucleotide substitution was used with the Gamma model of rate heterogeneity.

The *V. japonicum* plastome is 187,213 bp long, with two inverted repeat (IR) regions (39,788 bp each) that separate a large single copy (LSC) region (104,637 bp) and a small single copy (SSC) region (3000 bp). The genome has a pair of enlarged IR regions and an extremely shortened SSC region, which contains only a single gene—*ndh*F. In total, 135 genes, which include 88 protein-coding, eight ribosomal RNA, and 39 transfer RNA genes, were annotated. The GC content in overall, LSC, SSC, IR regions are 36.7%, 35.7%, 29.3%, and 38.4%, respectively. The most similar plastome, compared to *V. japonicum* (187,213 bp) in gene content and order, is *V. macrocarpon* (176,045 bp), despite the length difference.

The ML tree shows that all five *Vaccinium* species, *V. bracteatum*, *V. vitis-idaea*, *V. uliginosum*, *V. macrocarpon* and *V. japonicum*, are a monophyletic group with a 100% bootstrap value (Figure 1). This suggests that the complete plastome sequenced in this study is a valuable input to the genomic resources of Ericaceae and can be utilized in future evolutionary studies.

**Figure 1.**
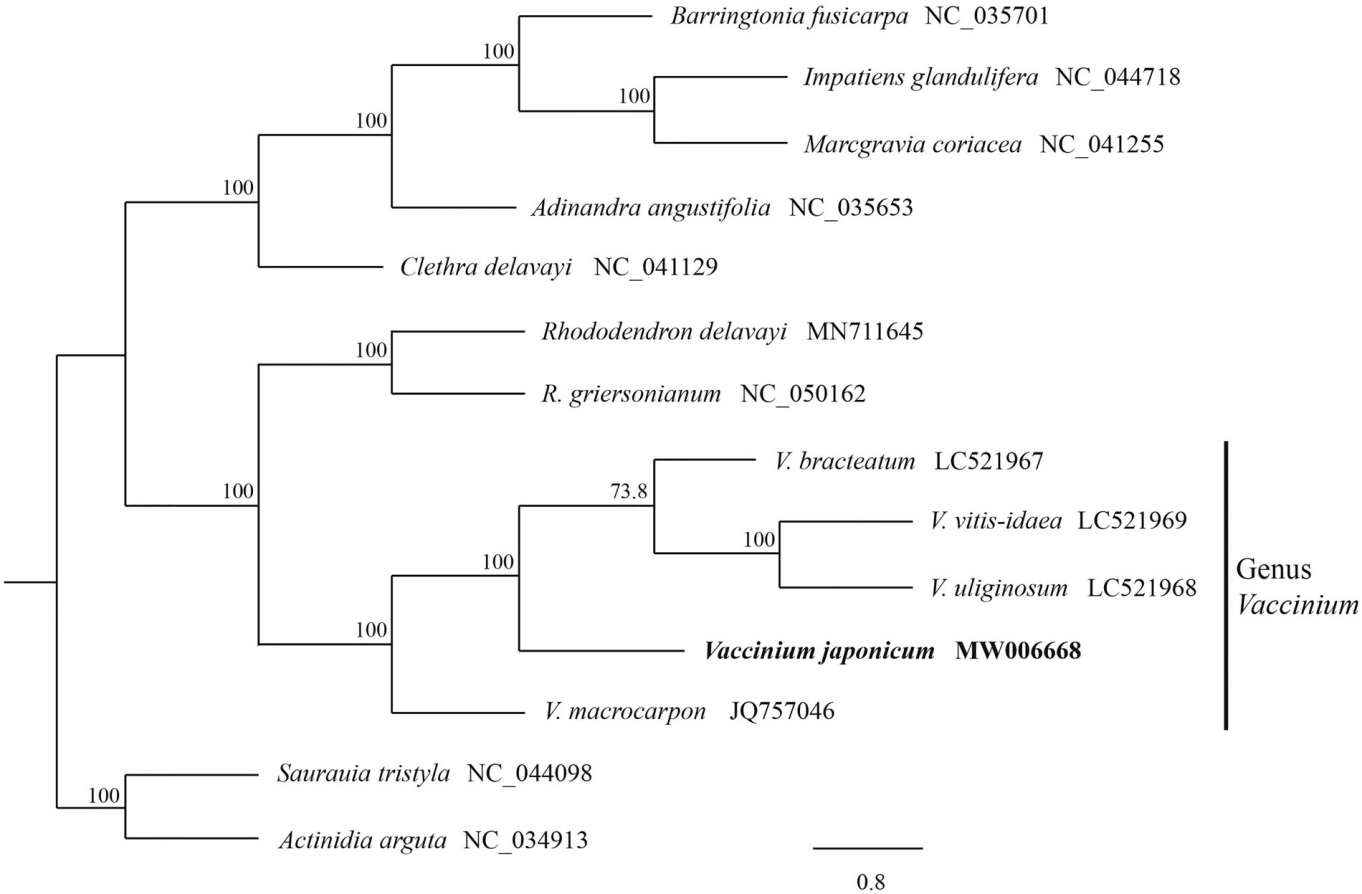
Maximum-likelihood phylogenetic tree constructed with RAxML using complete plastomes of the 14 species of Ericales. Numbers above the nodes indicate bootstrap values with 1000 replicates.

## Data Availability

The genome sequence data that support the findings of this study are openly available in GenBank of NCBI at [https://www.ncbi.nlm.nih.gov] (https://www.ncbi.nlm.nih.gov/) under the accession no. MW006668. The associated Sequence Read Archive (SRA) number is PRJNA681995.

## References

[CIT0001] Boufford DE, Ohashi H, Huang TC, Hsieh CF, Tsai JL, Yang KC, Ipeng C, Kuoh CS, Hsiao A. 2003. A checklist of the vascular plants of Taiwan. Flora Taiwan. 6:15–139.

[CIT0002] Dierckxsens N, Mardulyn P, Smits G. 2017. NOVOPlasty: de novo assembly of organelle genomes from whole genome data. Nucleic Acids Res. 45(4):e18. 28204566 10.1093/nar/gkw955 PMC5389512

[CIT0003] Fajardo D, Senalik D, Ames M, Zhu H, Steffan SA, Harbut R, Polashock J, Vorsa N, Gillespie E, Kron K, et al. 2013. Complete plastid genome sequence of *Vaccinium macrocarpon*: structure, gene content, and rearrangements revealed by next generation sequencing. Tree Genet Genomes. 9(2):489–498.

[CIT0004] Katoh K, Toh H. 2010. Parallelization of the MAFFT multiple sequence alignment program. Bioinformatics. 26:1899–1900. 20427515 10.1093/bioinformatics/btq224 PMC2905546

[CIT0005] Kearse M, Moir R, Wilson A, Stones-Havas S, Cheung M, Sturrock S, Buxton S, Cooper A, Markowitz S, Duran C, et al. 2012. Geneious basic: an integrated and extendable desktop software platform for the organization and analysis of sequence data. Bioinformatics. 28:1647–1649. 22543367 10.1093/bioinformatics/bts199 PMC3371832

[CIT0006] Kim SC, Baek SH, Lee JW, Hyun HJ. 2019. Complete chloroplast genome of *Vaccinium oldhamii* and phylogenetic analysis. Mitochondrial DNA B. 4(1):902–903.

[CIT0007] Kim Y, Shin J, Oh DR, Kim DW, Lee HS, Choi C. 2020. Complete chloroplast genome sequences of *Vaccinium bracteatum* Thunb., *V. vitis-idaea* L., *and V. uliginosum* L. (Ericaceae). Mitochondrial DNA Part B. 5(2):1843–1844.

[CIT0008] Kong WS, Kim KO, Lee SG, Park HN, Cho SH. 2014. Distribution of high mountain plants and species vulnerability against climate change. J Environ Impact Assess. 23(2):119–136.

[CIT0009] Lee TB. 2003. Illustrated flora of Korea. Seoul: Hyangmunsa; p. 25.

[CIT0010] Ruizheng F, Stevens PF. 2005. Vaccinium Linnaeus. Flora of China, Vol. 14. Beijing: Science Press & St. Louis: Missouri Botanical Garden Press; p. 476–504.

[CIT0011] Stamatakis A. 2014. RAxML version 8: a tool for phylogenetic analysis and post-analysis of large phylogenies. Bioinformatics. 30:1312–1313. 24451623 10.1093/bioinformatics/btu033 PMC3998144

[CIT0012] Tamada T. 2004. Blueberry production in Japan-today and in the future. VIII International Symposium on Vaccinium Culture 715, 267–272.

[CIT0013] Vander Kloet SP, Bohm BA. 1991. Taxonomy of *Vaccinium* section Oxycoccoides (Ericaceae). Rhodora. 93:226–237.

[CIT0014] Vander Kloet SP, Dickinson TA. 2009. A subgeneric classification of the genus *Vaccinium* and the metamorphosis of *V. section Bracteata* Nakai: more terrestrial and less epiphytic in habit, more continental and less insular in distribution. J Plant Res. 122(3):253–268. 19184674 10.1007/s10265-008-0211-7

[CIT0015] Yamazaki T. 1993. Ericaceae. In: Iwatsuki K, Yamazaki T, David EB, Ohba H, editors. Flora of Japan, Vol. IIIa. Tokyo: Kodansha; p. 6–63.

